# Antimicrobial activity of PMMA enriched with nano-clay loaded with metronidazole and chlorhexidine

**DOI:** 10.1590/1807-3107bor-2024.vol38.0110

**Published:** 2024-12-09

**Authors:** Eduardo Buozi Moffa, Samuel Santana Malheiros, Larissa Tavares Sampaio Silva, Delcio Ildefonso Branco, Regis Cléo Fernandes Grassia, William Cunha Brandt, Flavia Goncalves, Valentim Adelino Ricardo Barao, Letícia Cristina Cidreira Boaro

**Affiliations:** (a) University of Saskatchewan, College of Dentistry, Saskatchewan, Canada.; (b) Universidade Estadual de Campinas - Unicamp, Piracicaba Dental School, Department of Prosthodontics and Periodontology, Piracicaba, SP, Brazil.; (c) Universidade de São Paulo – USP, School of Dentistry, Department of Biomaterials, São Paulo, SP, Brazil.; (d) Universidade de Santo Amaro – Unisa, School of Dentistry, São Paulo, SP, Brazil.

**Keywords:** Acrylic Resins, Polymethyl Methacrylate, Nanoparticles, Anti-Bacterial Agents

## Abstract

Poly(methyl methacrylate) (PMMA) materials are highly susceptible to microbial colonization, predisposing patients to oral infections. To address this concern, we loaded PMMA samples with montmorillonite clay (MMT), a crystalline nanoparticle, in combination with chlorhexidine (CHX) or metronidazole (MET) targeting improved antimicrobial action. PMMA samples were prepared with or without MMT loaded with either CHX or MET, establishing the following groups: control (acrylic resin without the addition of nanoparticles), MMT/CHX (acrylic resin with 5% by weight of MMT loaded with CHX), and MMT/MET (acrylic resin with 5% by weight of MMT loaded with MET). Mechanical properties such flexural strength, flexural modulus, and Knoop hardness were evaluated using a universal testing machine. Antimicrobial efficacy was assessed via agar diffusion tests against *Enterococcus faecalis* and *Porphyromonas gingivalis*. The addition of MMT loaded with CHX did not affect the flexural strength and flexural modulus of PMMA compared to the control group (p > 0.05). However, MMT/MET reduced all mechanical properties of PMMA (p < 0.05). Both loaded-PMMA materials demonstrated antibacterial activity against *E. faecalis* but not against *P. gingivalis*. In conclusion, the incorporation of MMT/CHX into acrylic resin appears to be the most promising approach to combat microbial colonization while preserving PMMA mechanical properties. Future research should focus on optimizing material characteristics to enhance antimicrobial properties, paving the way for clinical applicability.

## Introduction

Poly(methyl methacrylate) (PMMA) stands as one of the most prevalent and versatile polymeric materials in the field of dentistry. It finds utility in various applications, including conventional denture base, provisional restorations, and maxillofacial and implant-retained prostheses fabrication.^1,2^ The widespread use of PMMA can be attributed to its good mechanical attributes, which confer resilience under the rigors of masticatory forces. Furthermore, PMMA exhibits satisfactory aesthetic properties and represents a cost-effective material in dental practice.^3^ However, upon exposure to the oral environment, PMMA becomes covered with a salivary pellicle rich in proteins that serve as active binding sites for microbial colonization.^3^

Consequently, acrylic resin bases are prone to colonization by microorganisms that leads to the biofilm formation.^4,5^ When close to host soft and hard tissues, these biofilms can foster the development of local and systemic infections by promoting a diverse microbial reservoir. The non-shedding niche created by PMMA materials can contribute to the development of stomatitis, dental caries, periodontal diseases, and peri-implantitis.^6^ Furthermore, the constant swallowing and aspiration of microorganisms from the biofilm can predispose immunocompromised patients to systemic infections, notably pneumonia, gastrointestinal infections, and endocarditis.^2^

The propitious environment for microbial aggregation on PMMA materials is directly attributable to their surface features and lack of intrinsic antimicrobial properties. Although mechanical and chemical cleaning protocols for PMMA materials are continually proposed, their efficacy is variable. Moreover, these cleaning methods may inadvertently contribute to material degradation, escalating surface roughness, liquid sorption, bacterial adhesion over time, and diminishing esthetic and mechanical properties.^7,8^ Consequently, researchers are redirecting their focus towards modifying PMMA materials to confer intrinsic antimicrobial properties, impeding microbial colonization while preserving surface integrity.

Various strategies employing antimicrobial polymers have been suggested to impede biofilm accumulation. Among these strategies, biocidal polymers, antimicrobial-releasing polymers, and biocide surface coatings are frequently used.^9^ For instance, methacryloyloxydecyl pyridinium bromide, a polymer biocide, exhibited robust antimicrobial effects against *Candida albicans* when incorporated into acrylic resin. However, it also promoted significant cytotoxicity.^10^ Similarly, different drug-releasing polymers incorporating metallic ions, such as silver nanoparticles, demonstrated promising antibacterial effects when integrated into PMMA materials. Nevertheless, these materials suffered from increased surface roughness and lacked release control, thereby hindering clinical application due to potential material staining and elevated microbial accumulation.^11–14^

Numerous drugs have undergone testing for incorporation into PMMA. In this study, we used two model drugs: chlorhexidine, a broad-spectrum antimicrobial, and metronidazole, an antibiotic primarily active against anaerobic bacteria. Although chlorhexidine has been proposed for incorporation into PMMA previously, the absence of a nanocarrier resulted in a significant decrease in material mechanical properties, increased surface roughness, and excessive and rapid release.^15^ Upon incorporation into PMMA, metronidazole demonstrated promising antibacterial efficacy; however, some cytotoxic effects were observed, underscoring the necessity for improved drug carriers.^16^

Consequently, a pressing need exists for a biocompatible and mechanically stable antimicrobial complex to be integrated into the PMMA matrix. In this context, montmorillonite clay (MMT) appears to be an interesting candidate owing to its good mechanical and biological properties. MMT, a type of clay mineral nanoparticle, exhibits a crystalline structure comprising intercalated lamellar layers featuring a central octahedral alumina structure flanked by successive layers of tetrahedral silica.^17^ These nanoscale alumino-silicate layers, featuring dimensions that span from 1 to 5 nm in thickness and 100 to 500 nm in diameter, enable MMT to effectively transfer and receive mechanical stress from PMMA, thereby enhancing material mechanical properties depending on its concentration.^17,18^

Additionally, MMT can be combined with a polymer to create a polymer/clay nanocomposite with enhanced performance. The presence of MMT in the nanocomposite facilitates the loading of organic molecules, including antimicrobials. MMT exhibits an affinity for bioactive molecules and regulates their release, thereby serving as a controllable drug-delivery system.^19–21^ Additionally, MMT has demonstrated efficacy as a drug-delivery system, for example, when combined with chitosan, enhancing drug encapsulation and promoting a slowed release from the nanocomposite.^20^ Therefore, this study aimed to evaluate the impact of MMT loaded with CHX or MET on the mechanical and antibacterial properties of PMMA-based materials.

## Methods

### Incorporation of MMT/CHX and MMT/MET nanoparticles into methyl methacrylate resin

**Figure 1:**
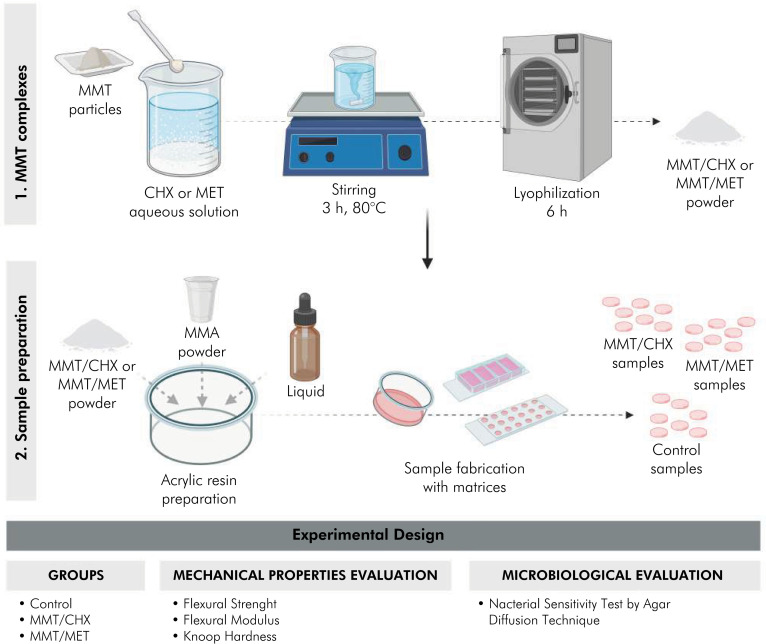
Schematic illustration of sample preparation and experimental design. Note: Control = acrylic resin samples without the addition of nanoparticles.

The incorporation of MMT/CHX and MMT/MET nanoparticles into chemically activated methyl methacrylate (MMA) composites was performed by adding montmorillonite clay (MMT) particles (Cloisite Southern Clay Products, Dallas, USA) to an aqueous solution of metronidazole (Sigma-Aldrich) or chlorhexidine (Sigma-Aldrich) in a weight ratio equivalent to 10% (w/w). The solution was stirred continuously for 3 hours at a temperature of 80°C. After the emulsion process, lyophilization was carried out for 6 hours.^19^ Subsequently, the MMT/MET or MMT/CHX complex was added to the PMMA powder (VIPI Flash, Pirassununga, Sao Paulo, Brazil) with an analytical balance and vigorously stirred for complete homogenization before manipulation. The concentration of MMT/Drug was established according to previous findings.^19^ Thus, three groups were established: control (acrylic resin without the addition of nanoparticles), MMT/CHX (acrylic resin with the addition of 5% by weight of MMT nanoparticles loaded with chlorhexidine), and MMT/MET (acrylic resin with the addition of 5% by weight of MMT nanoparticles loaded with metronidazole).^19^ Figure 1 summarizes the sample preparation process and the variables under investigation in this study.

MMT/CHX: acrylic resin samples with the addition of MMT nanoparticles loaded with chlorhexidine; MMT/MET: acrylic resin samples with the addition of MMT nanoparticles loaded with metronidazole; MMT: montmorillonite; CHX: chlorhexidine; MET: metronidazole; MMA: Polymethyl Methacrylate). Created with BioRender.com (License number: ZJ26LAW22X).

### Samples preparation

To fabricate the 36 specimens (n = 12/group) required for mechanical properties testing, matrices made of condensation silicone measuring 65 x 10 x 4 mm (length, width, and height, respectively) were utilized. Matrices were filled with self-cured acrylic resins under controlled temperature conditions of 25 ± 1°C. For the microbiological assay 30 specimens (n = 5/group) were fabricated using a condensation silicone circular matrix measuring 7 x 1 mm (diameter and thickness, respectively). Specimens were kept for 10 minutes before their removal from the matrix to standardize the final setting of the materials. Upon completion of specimen fabrication, they were stored in an incubator at 37°C for three days to allow for the release of any residual monomer. The specimens for the microbiological assay were disinfected with 70% alcohol.

### Mechanical properties

#### Flexural Strength (FS) and Flexural Modulus (FM)

To evaluate mechanical properties, we utilized a universal testing machine (Instron, 5565, Canton, USA) operating at a controlled speed of 1 mm/min according to previous studies.^22^ Before testing, all specimens underwent polishing with 600-grit sandpaper to ensure surface standardization. Subsequently, the three-point flexural strength test was conducted to assess the material's resistance to bending forces. The span distance was 50 mm according to ISO 20795-1.^23^ The equation below was utilized to calculate the maximum flexural strength (FS):


$$\sigma =\frac{3FL}{2bh^{2}}$$

where, σ represents the flexural strength (MPa), F denotes the maximum load registered prior to specimen fracture (N), L signifies the distance between the supports (mm), b indicates the width of the specimen (mm), and h represents the height of the specimen (mm). Flexural modulus (FM) was calculated as follows:


$$E=\frac{CL^{3}}{4bh^{3}D}10^{-3}$$

where, E represents the flexural modulus (Gpa), C denotes the recorded load (N), L signifies the span between the supports (mm), b indicates the width of the specimen (mm), h represents the height of the specimen (mm), and D stands for the deflection corresponding to C (mm). The flexural modulus was determined from the linear region of the stress/strain curve, employing the BlueHill Universal software integrated within the testing apparatus.

#### Knoop hardness

Following sample fracture, the larger broken segment was selected for Knoop hardness testing. Using an indenter (MV-2, Shimadzu, Tokyo, Japan) under a 50 g load for 10 seconds, Knoop hardness measurements were conducted. Five readings were obtained per sample, with the Knoop hardness number (KHN, Kg/mm²) derived from averaging the five indentations.^24^

### Antibacterial activity

#### Growing conditions

*Porphyromonas gingivalis* and *Enterococcus faecalis* strains were used for this analysis. Prior to the experiment, a bacterial suspension was prepared in Agar Blood for *P. gingivalis* and Tryptic soy broth for *E. faecalis* for 24 hours. Adjustments of each culture were carried out separately by measuring their absorbance at 625 nm (optical density = 0.1) with a spectrophotometer.

#### Bacterial sensitivity test by agar diffusion technique

The agar diffusion technique was performed following the recommendations of the CLSI – *Clinical and Laboratory Standards Institute* (Performance Standards for Antimicrobial Disk Susceptibility Tests: Approved Standard – Eighth Edition, M02-A12).^25^ Petri dishes containing Blood Agar or Tryptic Soy Broth were inoculated with the respective bacterial suspensions. For each group, specimens (n = 5/group) were placed on the surface of the seeded agar plate. *E. faecalis* plates were kept in an incubator at 37°C, and *P. gingivalis* plates were kept in the same incubator but under anaerobic conditions, for 18 h.^26^ After the incubation period, the dimensions of the entire inhibition zones were measured using a digital caliper recorded in millimeters. The inhibition halo was characterized by the absence of discernible growth to the naked eye.^27^

### Statistical analysis

Data was analyzed using IBM SPSS Statistics for Windows (v.21.0., IBM Corp., Armonk, US). The Shapiro-Wilk method was utilized to assess the normality, while the Levene test was employed to examine the homoscedasticity of the data. One-way analysis of variance (ANOVA) was performed on flexural strength, flexural modulus, and Knoop hardness test data, with acrylic resin composition as the independent variable. Post hoc multiple comparisons were conducted using the Tukey honestly significant difference (HSD) test. Student's T-Test was applied to microbiological data to ascertain differences between the two experimental groups. A significance level of 5% was utilized for all analyses. Graphs were generated using GraphPad Prism version 8.0.0 for Windows (GraphPad Software, San Diego, USA).

## Results

**Figure 2:**
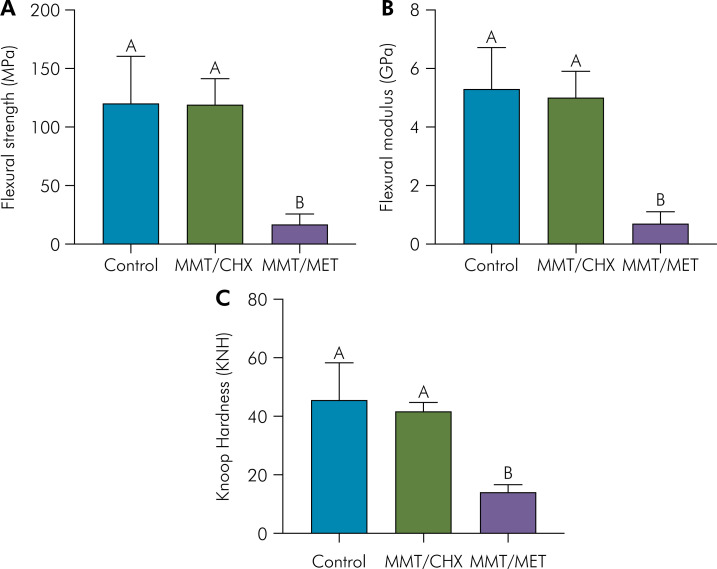
Mean values and standard deviations of a) flexural strength, (b) flexural modulus, and (c) Knoop hardness (KH) of acrylic resins samples of each group.

MMT: montmorillonite; CHX: chlorhexidine; MET: metronidazole)Statistically significant differences between groups are denoted by distinct capital letters (p < 0.05).

The results of the flexural strength, flexural modulus, and Knoop hardness tests are summarized in Figure 2. No difference in mechanical properties (flexural strength, flexural modulus, and Knoop hardness) between the group treated with chlorhexidine (119.2 MPa; 5.0 GPa; 41.8 KHN) and the control group (120.2 MPa; 5.3 GPa; 45.5 KHN). However, there was a notable decrease in flexural strength, flexural modulus, and Knoop hardness when metronidazole, in conjunction with MMT particles, was added to the polymer matrix (16.9 MPa; 0.7 GPa; 14.1 KHN).

**Figure 3:**
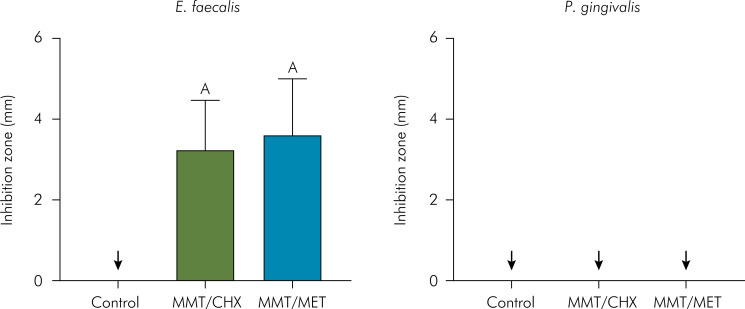
Mean values and standard deviations of the inhibition zones in mm for the tested control and experimental groups against *E. faecalis* and *P. gingivalis*.

MMT: montmorillonite; CHX: chlorhexidine; MET: metronidazole Statistically significant differences between groups are denoted by distinct capital letters (p < 0.05). Arrows in the graph indicates that no inhibition zones were observed across the samples, suggesting no antimicrobial activity against the tested bacterial strains.

Data from the bacterial sensitivity test using the agar diffusion technique are shown in Figure 3. Both chlorhexidine and metronidazole demonstrated antimicrobial activity against *E. faecalis*. Although no statistically significant difference in the inhibition zones for *E. faecalis* was observed between the two drugs (t-test, p > 0.05), both exhibited positive antibacterial effects. However, neither chlorhexidine nor metronidazole showed any inhibition zones against *P. gingivalis* when incorporated into the polymer-MMT complex. Similarly, no inhibition zones were observed in the control group for both bacterial strains.

## Discussion

Acrylic resins are extensively utilized in dentistry for various applications, including the fabrication of prostheses, orthodontic appliances, and temporary indirect restorations. Their utilization can be mainly attributed to their affordability and satisfactory mechanical and aesthetic characteristics, making them versatile for various clinical applications.^28^ However, a significant limitation is their susceptibility to microbial colonization and lack of intrinsic antimicrobial effect.^5,29,30^ The present study evaluated the mechanical and antimicrobial properties of PMMA materials incorporated with MMT loaded with CHX or MET. Our findings shed light on several key aspects regarding the feasibility and efficacy of this novel approach.

Regarding mechanical properties, adding CHX-loaded MMT into the PMMA matrix did not significantly alter the flexural strength, flexural modulus, or Knoop hardness of the samples compared to the control group. This suggests that CHX incorporation within the studied parameters did not compromise the mechanical integrity of the PMMA matrix. In accordance, a recent study also showed that the addition of nanoparticles loaded with chlorhexidine in a resin matrix does not promote significant mechanical properties alteration, thereby suggesting the inertness of CHX on polymeric matrixes.^22^ Despite MMT's crystalline structure, the absence of improvement in the mechanical strength of the samples indicates that the low concentration of MMT utilized served primarily as a drug delivery vehicle rather than reinforcement for the PMMA matrix.^19^

Conversely, including MET-loaded MMT resulted in a significant decrease in all tested mechanical properties. This observation indicates a detrimental effect of MET on the structural integrity of the PMMA matrix. Interestingly, a previous study suggested that low MET concentrations could prolong the polymerization process of PMMA for approximately 48 hours,^31^ which in turn could affect the material's properties. Also, it has already been verified that when nanoparticles are added to the resin matrix and present a limited interaction with the polymer, forces can induce structural disorganization within the nanocomposite, decreasing flexural strength^13^. These previous findings can help clarify the negative effect of MET on mechanical properties of PMMA materials.

In contrast to studies predominantly employing *C. albicans* to evaluate the antimicrobial efficacy of their experimental resins, the current investigation focused on the bacterial strains *P. gingivalis* and *E. faecalis*. Among the 700 bacterial species colonizing the oral environment^32^, *E. faecalis* stands out for its association with persistent odontogenic infections and endodontic treatment failures, which can be explained by its resilience in adverse conditions, such as nutrient scarcity and high alkalinity.^6,33–35^ Additionally, *P. gingivalis* holds significance in the field of dental pathogens, being recognized as a primary contributor to the development of periodontitis.^32,36^ Considering the intimate contact between acrylic resin and the supporting dental tissues, it is reasonable to hypothesize that incorporating bactericidal agents into the polymeric matrix could potentially mitigate the progression or onset of periodontal disease. Hence, the selection of these microorganisms for assessing the antimicrobial efficacy of chlorhexidine and metronidazole appears advantageous, as successful outcomes could potentially be extrapolated to combat other, less resistant microbial strains prevalent in the oral cavity.

The inhibition zones observed in agar diffusion tests demonstrated that both MMT/CHX and MMT/MET have antimicrobial activity against *E. faecalis*. These findings suggest that CHX and MET were released from the composite into the agar medium, preventing bacterial growth. Metronidazole acts against *E. faecalis* by penetrating the bacterial cell, undergoing reduction of its nitro group, leading to the formation of cytotoxic intermediates. These intermediates then target essential biomolecules such as RNA, DNA, or cellular proteins, ultimately resulting in bacterial cell death.^37^ Chlorhexidine has demonstrated promising results when incorporated into resin matrices alongside a carrier, reducing different bacteria species viability, such as *Streptococcus mutans*, *Streptococcus mitis*, *Streptococcus gordonii*, and *Staphylococcus aureus.* ^19,22^ Chlorhexidine induces cell membrane disorganization and leakage of cytoplasmatic components producing the coagulation of microorganisms’ cytoplasmatic constituents.^28^ However, surprisingly, neither MET nor CHX exhibited inhibitory effects against *P. gingivalis*. This finding can be attributed to the bacterium's inherent higher resistance to damage to its proteins, lipid layer, and DNA, coupled with its exceptional DNA repair capabilities.^38^ These mechanisms suggest that a direct contact mechanism may be necessary for effective antimicrobial action rather than solely drug release.

It is important to acknowledge the limitations of this study. The experimental design focused on assessing mechanical and antimicrobial properties *in vitro*. Further studies are warranted to evaluate the long-term stability, biocompatibility, and clinical efficacy of these modified PMMA materials. Additionally, exploring alternative drug carriers or adjusting drug loading concentrations may offer opportunities to optimize the formulation and enhance therapeutic outcomes.

## Conclusion

In conclusion, incorporating chlorhexidine-loaded montmorillonite clay into PMMA materials shows promise in maintaining mechanical integrity while imparting antimicrobial properties against specific bacterial strains. However, further research is needed to optimize the formulation and validate its clinical efficacy in preventing microbial colonization and associated oral infections.
